# The protein interaction of mitochondrial transcription factors A and B2 is associated with 30-day survival in critical COVID-19

**DOI:** 10.3389/fimmu.2025.1445403

**Published:** 2025-05-16

**Authors:** Britta Westhus, Patrick Thon, Lars Palmowski, Katharina Rump, Björn Koos, Frederik Wiebel, Birte Dyck, Martin Eisenacher, Barbara Sitek, Stephanie Pfaender, Nina Babel, Moritz Anft, Christian Putensen, Stefan Felix Ehrentraut, Christina Weisheit, Alexander Zarbock, Thilo von Groote, Andrea Witowski, Matthias Unterberg, Hartmuth Nowak, Alexander Wolf, Lars Bergmann, Michael Adamzik, Dominik Ziehe, Tim Rahmel

**Affiliations:** ^1^ Klinik für Anästhesiologie, Intensivmedizin und Schmerztherapie, Universitätsklinikum Knappschaftskrankenhaus Bochum, Bochum, Germany; ^2^ Medical Proteome Analysis, Center for Proteindiagnostics (PRODI), Ruhr-University Bochum, Bochum, Germany; ^3^ Medizinisches Proteom-Center, Ruhr-University Bochum, Bochum, Germany; ^4^ Department of Molecular and Medical Virology, Ruhr-University Bochum, Bochum, Germany; ^5^ Leibniz-Institut für Virologie, Hamburg, Germany; ^6^ Center for Translational Medicine, Medical Clinic I, Marien Hospital Herne, University Hospital of the Ruhr-University Bochum, Herne, Germany; ^7^ Klinik für Anästhesiologie und Operative Intensivstation, Universitätsklinikum Bonn, Bonn, Germany; ^8^ Klinik für Anästhesiologie, operative Intensivmedizin und Schmerztherapie, Universitätsklinikum Münster, Münster, Germany; ^9^ Zentrum für Künstliche Intelligenz, Medizininformatik und Datenwissenschaften, Bochum, Germany

**Keywords:** COVID-19, mitochondrial dysfunction, TFAM, mitochondrial biogenesis, protein interaction, proximity ligation assay

## Abstract

**Introduction:**

Repair of mitochondrial damage seems pivotal for clinical recovery and determining outcome in patients with critical COVID-19. However, reliable biomarkers for non-invasively assessing mitochondrial repair in peripheral blood of critically ill COVID-19 patients are currently lacking. Accordingly, we sought to assess different surrogates of mitochondrial repair in peripheral blood and correlate these measurements with clinical outcome in patients with critical COVID-19.

**Methods:**

In this prospective multicentric cohort study, 88 critically ill COVID-19 patients were enrolled across three German intensive care units. Gene products of mitochondrial quality control (MFN2, PINK, TFAM, TFB2M) and the mtDNA copy number were measured in peripheral blood mononuclear cells. Furthermore, the protein interactions between TFAM and TFB2M were quantified. Patients were stratified regarding 30-day mortality.

**Results:**

Transcript levels of the assessed mRNA markers of mitochondrial quality control were not associated with clinical outcome. In contrast, more than 10.7 protein interactions per cell were associated with a 74% 30-day survival (37 out of 50), while 10.7 or fewer protein interactions per cell were associated with a 32% 30-day survival (12 out of 38; p < 0.001). Furthermore, multivariable Cox regression analysis revealed TFAM-TFB2M protein interaction as an independent predictor for 30-day survival (HR: 3.2; 95% CI: 1.6 to 6.5; p < 0.001).

**Discussion:**

Our findings indicate that TFAM-TFB2M protein interactions, identified as a novel biomarker, are strongly and independently associated with 30-day survival in critical COVID-19. Therefore, our data suggest a significant impact of mitochondrial repair and quality control on clinical outcome in critical COVID-19.

## 1 Introduction

An infection with the SARS-CoV-2 virus can result in COVID-19 with a varying severity of clinical manifestations, from mild to critical illness. Especially critical COVID-19 requires intensive care treatment, potentially leading to long-term sequelae and death (1, 2). The predominant manifestation of critical COVID-19 is respiratory failure, septic shock, and multiple organ dysfunction, mimicking the characteristics of bacterial sepsis (3, 4). In both bacterial sepsis and critical COVID-19, multi-organ failure is affected by mitochondrial dysfunction (4, 5). Here, previous studies have demonstrated that SARS-CoV-2 induces mitochondrial structural changes and damage, resulting in mitochondrial dysfunction (6–10). Accordingly, mitochondrial repair via processes of mitochondrial quality control (MQC), seems pivotal for functional recovery likely impacting clinical outcome in COVID-19 (11–14).

Previous studies in sepsis patients already demonstrated that messenger RNA (mRNA) transcripts in peripheral blood mononuclear cells (PBMCs) related to MQC, especially mitochondrial biogenesis, represent potential biomarker candidates, which were able to display clinical outcome in septic patients (15). A further promising candidate to assess mitochondrial repair in patients suffering from COVID-19 is the interaction between mitochondrial transcription factor A (TFAM) and mitochondrial transcription factor B2 (TFB2M) (16). TFAM and TFB2M are crucial components of the mitochondrial transcription initiation complex, essentially promoting mitochondrial biogenesis (17, 18). In bacterial sepsis, a reduced number of protein interactions between TFAM and TFB2M measured in PBMCs was associated with inefficient mitochondrial recovery and increased mortality in sepsis (19). To determine the complex formation between TFAM and TFB2M in critical COVID-19, a well-established assay, called proximity ligation assay (PLA), was performed (35). The PLA is a fluorescence-based method that is used to measure protein-protein interactions or protein modifications *in situ*. PLA signals are only detected when two proteins are in close proximity to one another, which is therefore a measure of the ability of two proteins to interact.

Thus, the objective of this study is to investigate different mRNA transcripts related to mitochondrial repair and quality control including the protein interaction between TFAM and TFB2M targeting mitochondrial biogenesis, as predictors for clinical outcome in patients with critical COVID-19.

## 2 Study design and methods

### 2.1 Study design and population

This multicentric prospective cohort study was conducted on three intensive care units (ICUs) of German university hospitals from September 2020 to December 2021 as part of CovidDataNet.NRW (German Clinical Trial Registry No. DRKS00026184). The study was approved by the Ethics Committee of the Medical Faculty of the Ruhr-University Bochum (registration number #5047-14), and subsequently by the local ethics committees of each participating center. Written informed consent was secured from all participants or their legal representatives prior to enrolment. This study was conducted in strict accordance with the Declaration of Helsinki, guidelines for good clinical practice, and relevant local legal regulations.

Patients with critical COVID-19 were considered eligible if the infection with SARS-CoV-2 was verified using polymerase chain reaction (PCR) assays and if they fulfilled the criteria for critical course of COVID-19 defined according to the World Health Organization as a disease with the confirmation of one (or more) of the following symptoms:

- Respiratory distress ≥ 30 breaths per minute.
- Oxygen saturation ≤ 93% at rest under ambient air or mandatory oxygen.
- Oxygenation index ≤ 300 mmHg [arterial oxygen partial pressure (paO_2_)/fractional inspired oxygen (FiO_2_)].
- Presence of respiratory failure, septic shock, and/or multiple organ dysfunction.

Furthermore, the initial blood sampling was required to be completed within the first 24 hours after the diagnosis of sepsis. The exclusion criteria were age under 18 years, pregnancy, existing anemia, a diagnosed mitochondrial disorder, usage of medications identified as mitochondrial toxins, and rejection of the study by the patient or legal representatives. In total 88 adult patients diagnosed with critical COVID-19 were enrolled between September 2021 and December 2022.

### 2.2 Procedures/exposure

#### 2.2.1 Sample preparation

Whole blood samples were collected in sodium EDTA tubes (BD Biosciences, Heidelberg, Germany) on the day of enrollment. PBMCs and plasma fractions were isolated using Ficoll density gradient centrifugation (GE Healthcare Europe, Freiburg, Germany). For microscopy the cells were counted, spun onto microscope slides using a cytospin (Cellspin II, Tharmac, Wiesbaden, Germany) and then fixed with 4% formaldehyde solution (Sigma-Aldrich). PBMCs, dissolved in Bambanker freezing medium (Nippon Genetics, Düren, Germany), and plasma were stored at -80°C.

#### 2.2.2 Quantitative polymerase chain reaction

To investigate, gene products associated with MQC in critical COVID-19, quantitative polymerase chain reaction (qPCR) was performed. mRNA transcripts of mitofusion 2 (MFN2), PTEN-induced kinase 1 (PINK1), TFAM and TFB2M as well as the copy number of mitochondrial DNA (mtDNA) were assessed. Therefore, total DNA and total RNA were extracted from PBMCs using the QIAamp DNA and RNeasy kits according to the manufacturer’s instructions (QIAGEN, Hilden, Germany). The synthesis of complementary DNA (cDNA)-synthesis was performed using the High-Capacity cDNA Kit (Applied Biosystems, Waltham, MA, USA). PCR was conducted in technical duplicates using GoTaq^®^ qPCR Master Mix (Promega, Walldorf, Germany) and specific primers (**Table 1**) on a CFX Connect Real-Time System (Bio-Rad Laboratories, Feldkirchen, Germany). The relative mRNA expression level and mtDNA copy number quantification was performed as previously described (16, 19).

**Table 1:** Primer sequences of selected genes products (from Integrated DNA Technologies) associated with mitochondrial function and vitality as well as primer sequences of control gene products.

Gene name	Sequence (5`-3`)		Efficiency
ACTB (beta actin)	forward	CCTTCCTGGGCATGGAGT	105.80% (->100%)
	reverse	CAGGGCAGTGATCTCCTTCT	
MFN2 (Mitofusin 2)	forward	TGATGGGCTACAATGACCAG	102.60% (-> 100%)
	reverse	AGCTTCTCGCTGGCATGC	
TFAM (Mitochondrial transcription factor A)	forward	CAGAACCCAGATGCAAAAACT	105.50% (-> 100%)
	reverse	TGTCCATGATTTCTTTTTCCAA	
TFB2M (Mitochondrial transcription factor 2B)	forward	CTGGAAAACCCAAAGCGTAG	111.70% (-> 100%)
	reverse	CTCGCATCAAGTGGAGTCAA	
PINK1 (PTEN induced kinase 1)	forward	GGGGAGTATGGAGCAGTCAC	108.00% (-> 100%)
	reverse	CATCAGGGTAGTCGACCAGG	
mtND1 (Mitochondrial NADH dehydrogenase subunit 1)	forward	CACCCAAGAACAGGGTTTGT	91.00%
	reverse	TGGCCATGGGTATGTTGTTAA	
18S-rRNA (18S ribosomal RNA)	forward	TAGAGGGACAAGTGGCGTTC	54.00%
	reverse	CGCTGAGCCAGTCAGTGT	

#### 2.2.3 Proximity ligation assay

The PLA is used to measure protein-protein interactions *in situ*. If two proteins interact with each other, signals occur in the subsequent PLA reaction. Only when two proteins are in close proximity to one another signals in the form of individual dots could be detected. Therefore, PLA signals per cell are a measure of the ability of two proteins to interact. To analyze the protein-protein interaction of TFAM and TFB2M, a proximity ligation assay (PLA) was performed as previously described [16]. Slides were re-hydrated, permeabilized and primary antibodies against TFAM (1:50, sc-376672, Santa Cruz Biotechnology, Heidelberg, Germany) and TFB2M (1:100, 46-481, ProSci Incorporated, Poway, CA, USA) were incubated overnight at 4°C. After additional washing steps, an incubation with secondary antibodies (anti-mouse and anti-rabbit, NaveniFlex 100 MR, Navinci Diagnostics, Uppsala, Sweden) was following for 1 hour at 37°C. The subsequent PLA protocol was performed according to the manufacturer’s recommendations (Navinci Diagnostics). PBMCs were imaged using Zeiss Software ZEN for a Zeiss Apotome microscope (Zeiss, Oberkochen, Germany). Images were analyzed using ImageJ (Version 1.8.0_345) and CellProfiler (Version 4.2.3.) to quantify the average PLA signal per cell. For an appropriate analysis, at least 50 PBMCs were needed per patient. Out of 95 samples, 88 were analyzed.

### 2.3 Data collection and variables

Data were sourced from electronic health records, transferred to a central database, and verified at each study site. The dataset was validated by an independent intensive care specialist and a statistician for accuracy. Any discrepancies were addressed by local teams during data clearance.

### 2.4 Outcomes

For the evaluation of the association with clinical outcome, we stratified sepsis patients into two clinical outcome groups defined as “30-day survivors” (n = 49) and “30-day non-survivors” (n = 39). Given the hypothesis that the severity of COVID-19 upon ICU admission is associated with the degree of mitochondrial dysfunction at that time, we evaluated the Sepsis-related organ failure assessment score (SOFA score), using the protocol described by Lambden and colleagues (20), on the first day simultaneously with blood collection.

### 2.5 Statistical analysis

Severity scores for diseases were expressed as the median and the interquartile range (IQR). Variables on an interval scale with normal distribution were depicted as mean ± standard deviation (SD), alongside median and IQR (25th to 75th percentiles) as appropriate. Categorical variables were presented as counts and percentages. Confidence intervals (CIs) were established at a 95% level. All p-values are nominal, two-sided, and were considered significant at a predefined level of less than 0.05. The normality of the distribution within each group was assessed using the Shapiro-Wilk test. Consequently, differences between two normally distributed groups were analyzed using the unpaired Student’s t-test, while differences between two groups not following a normal distribution were evaluated using the Mann-Whitney U test.

Further analysis involved determining the cut-off for TFAM-TFB2M interaction based on the Youden Index, to distinguish between high (>10.7 PLA signals per cell) and low (≤10.7 PLA signals per cell) protein activity. This was followed by Kaplan-Meier survival analysis and Cox regression models to evaluate the impact of mitochondrial protein interactions on the first day on survival over 30 days. Initially, a univariate Cox regression analysis was performed using TFAM-TFB2M protein interactions as variable, stratified by the Youden Index threshold. A multivariate Cox regression model was then performed, additionally considering adjustment for age and the SOFA score. Statistical analyses were carried out using SPSS software (version 28, IBM, Chicago, IL, USA), while GraphPad Prism 9 (GraphPad, San Diego, CA, USA) facilitated the creation of graphical representations.

## 3 Results

### 3.1 Patient characteristics

Ninety-five patients with severe COVID-19 on three different ICUs were enrolled in this study, but the final analysis was conducted on 88 patient (**Table 2**). The mean age was 58 (± 1.6) years and median SOFA score was 9 [5 to 12]. Further baseline characteristics are shown in **Table 2**.

**Table 2:** Baseline characteristics of patients with critical COVID-19.

	30-day survivors (n=49)	30-day non-survivors (n=39)	P value
Demographics			
Age [years], mean ( ± SD)	56.25 ( ± 14.4)	61.5 ( ± 16.7)	0.113
Female sex, n (%)	19 (39)	15 (38)	0.976
Immunisation status			
Vaccinated, n (%)	12 (25)	6 (15)	0.352
Unvaccinated, n (%)	22 (45)	19 (49)	
COVID-19-variant			
Alpha, n (%)	8 (16)	2 (5)	0.369
Delta, n (%)	14 (29)	11 (28)	
Omicron, n (%)	8 (16)	9 (23)	
unknown, n (%)	18 (37)	17 (44)	
Medical history, n (%)			
Cardiovascular comorbidities	11 (22)	9 (23)	0.944
Hypertension	24 (49)	22 (56)	0.488
Pulmonary comorbidities	7 (14)	7 (18)	0.641
COPD*	45 (91)	39 (100)	0.068
Diabetes mellitus	19 (38)	7 (18)	0.033
Obesity	26 (53)	13 (33)	0.064
Malignant disease	1 (2)	6 (15)	0.022
Status at ICU admission (first 24h)			
SOFA score**; median (IQR)	8 [3.5 to 12]	10 [7.8 to 13.3]	0.033
SAPS II ***; median (IQR)	38 [26.5 to 48]	47 [35.8 to 55]	0.024
Mechanical ventilation, n (%)	16 (32)	18 (46)	0.058
Vasopressor support, n (%)	16 (32)	21 (53)	0.003
Laboratory values at ICU admission (first 24h); median (IQR)			
Leukocyte count [1000/µL]	10.7 [6.1 to 18.2]	10.6 [6.5 to 18.4]	0.023
C reactive protein [mg/L]	13.8 [7.8 to 27.6]	10.7 [4 to 16.6]	0.676
Procalcitonin [ng/mL]	0.5 [0.1 to 1.5]	0.3 [0.2 to 0.7]	0.143
Total bilirubin [mg/dL]	0.6 [0.4 to 0.8]	0.6 [0.4 to 1.2]	0.029
ALAT^§^ [U/L]	28.8 [15.7 to 82.9]	52.5 [29.2 to 101.1]	0.236
ASAT^ll^ [U/L]	46.7 [27.4 to 83.8]	67 [39 to 128.3]	0.118
Platelet count [1000/µL]	274 [137 to 304]	216 [146 to 291]	0.387
Serum creatinine [mg/dL]	1.1 [0.7 to 1.7]	0.9 [0.7 to 1.4]	0.609
Serum lactate [mmol/L]	1.55 [1.23 to 2.0]	2.6 [2.1 to 4.4]	< 0.001
D-Dimere [µg/mL]	3787 [1032 to 28483]	5694 [312 to 74562]	0.880
CMV^‡^ -IgG-Status positive, n (%)	33 (67)	27 (69)	0.414
CMV-IgG-Status negative, n (%)	10 (20)	5 (13)	

*Chronic Obstructive Lung Disease; **Sequential Organ Failure Assessment score; ***Simplified Acute Physiology Score; ^§^Alanine-Aminotransferase; ^II^Aspartate-Aminotransferase; ^‡^CMV, Cytomegalovirus.

### 3.2 30-day survivors versus 30-day non-survivors

**Figure 1:**
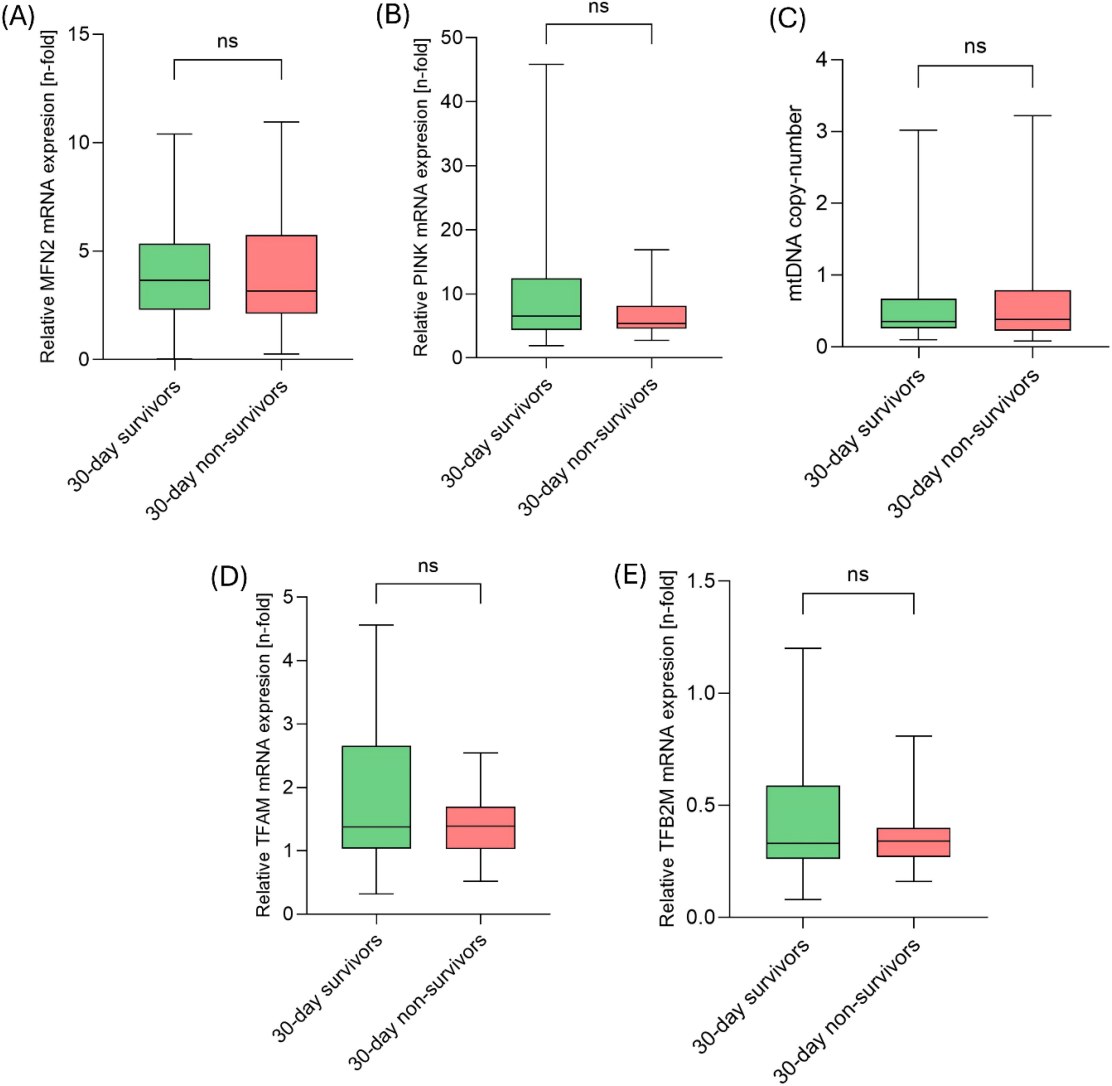
mRNA transcripts of MQC-related genes did not differ between 30-day survivors and 30-day non-survivors on day 1. **(A)** Comparison of relative MFN2 mRNA expression, **(B)** relative PINK mRNA expression, **(C)** mtDNA copy-number, **(D)** relative TFAM mRNA expression and **(E)** relative TFB2M mRNA expression between 30-day survivors and 30-day non-survivors.

First, we explored the role of different gene transcripts related to MQC. Here, mRNA-expression of MFN2, PINK, TFAM, and TFB2M along with the mtDNA copy number were assessed. However, no significant differences in gene expression were observed between the 30-day survivors and the 30-day non-survivors (each, p > 0.05; **Figure 1**).

**Figure 2:**
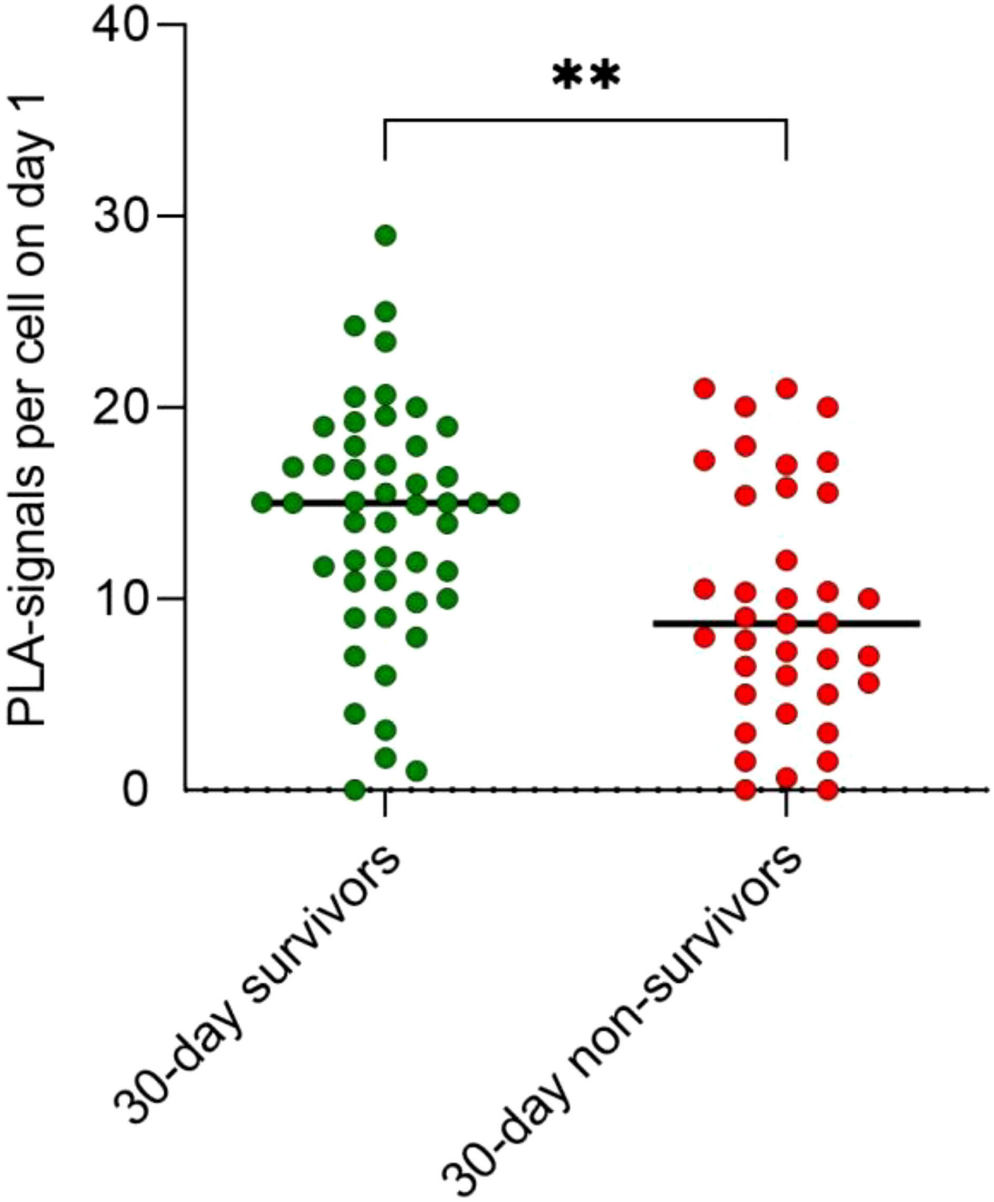
Interactions of TFAM with TFB2M in the human mitochondrial transcription initiation complex increases in 30-day survivors compared to 30-day non-survivors (** p = 0.0017).

In contrast, the mitochondrial protein interaction of TFAM with TFB2M within the mitochondrial transcription complex was increased on day 1 in 30-day survivors compared to 30-day non-survivors (p = 0.001; **Figure 2**). In detail, 44% higher quantity of PLA signals per cell were detected in 30-day survivors (15 signals per cell, IQR: 10.5 to 18.0) in comparison to 30-day non-survivors (8.7 signals per cell, IQR: 5.0 to 15.6).

### 3.3 Outcome of COVID-19

**Figure 3:**
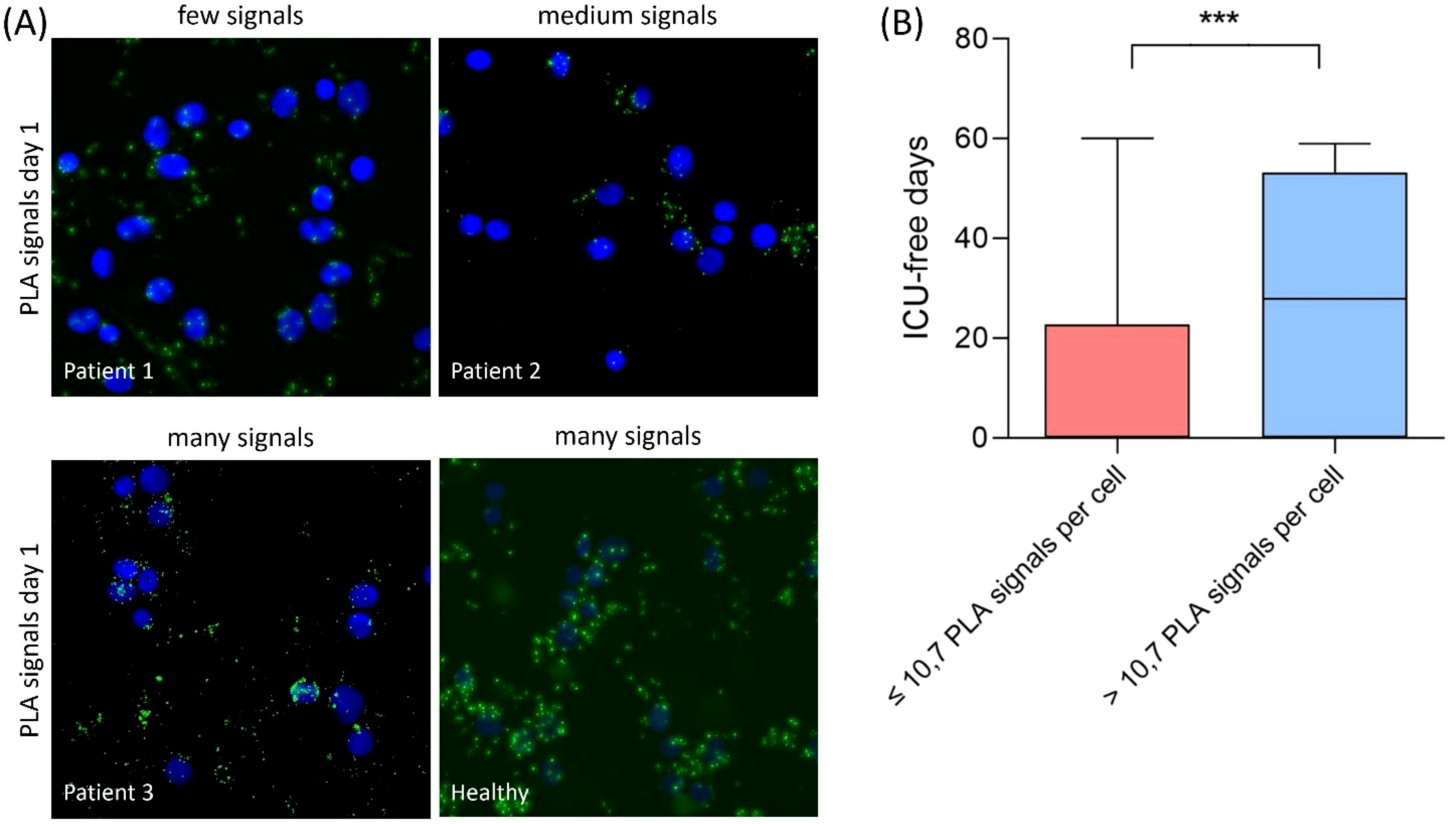
**(A)** Proximity ligation assay (PLA) in PBMCs of critical COVID-19 patients on day 1 and healthy control. PLA was conducted to assess the interaction between TFAM and TFB2M within the human mitochondrial transcription initiation complex, serving as an indicator of mitochondrial functionality and viability. Each green dot represents a signal for a TFAM/TFBM interaction. Nuclear staining with DAPI (blue) can be used to determine the average PLA signal per cell. The average PLA signal per cell was then associated with 30-day survival of critical COVID-19. **(B)** Patients with >10.7 PLA signals experience more ICU-free days over a 60-day period compared to patients with <10.7 PLA signals (*** p = 0.0008).

**Figure 4:**
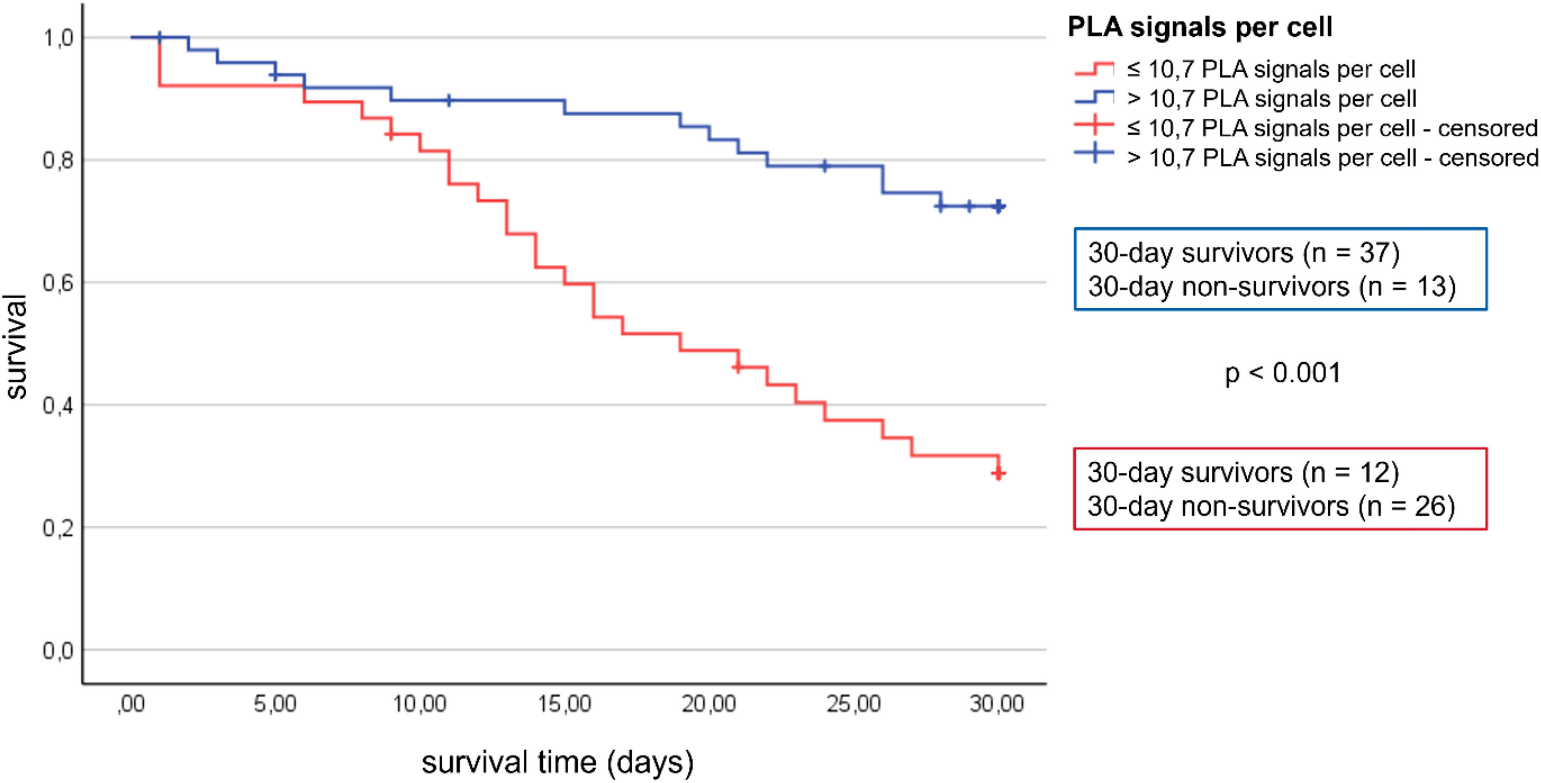
Patients with >10.7 PLA signals have a higher 30-day survival rate than patients with <10.7 PLA signals.

The interaction between TFAM and TFB2M was measured using proximity ligation assay (PLA) and the average PLA signal per cell was determined for each patient. There were differences in the average PLA signal per cell between the individual patients (**Figure 3A**). Therefore, a cut-off for TFAM-TFB2M protein interactions on day 1 regarding prediction of 30-day mortality was calculated using the Youden index. The area under the receiver operating characteristic curve was 0.72 (95% CI: 0.59 to 0.84; p = 0.002), with a sensitivity of 75% and a specificity of 67%. Correspondingly, more than 10.7 PLA signals per cell (high activity of mitochondrial biogenesis) were associated with a 74% survival rate at 30 days (37 of 50; **Figure 4**) and less than 10.7 PLA signals per cell (reduced activity of mitochondrial biogenesis) were related with a 32% survival rate at 30 days (12 of 38; p < 0.001; **Figure 4**). A multivariable Cox regression analysis revealed that a low amount of mitochondrial protein interactions of TFAM with TFB2M (≤ 10.7 PLA signals per cell) was associated with a more than 3-fold higher risk of death within 30 days after adjusting for age and SOFA score (hazard ratio: 3.3; 95% CI: 1.6 to 6.5, p < 0.001; **Table 3**). Thus, Cox regression analyses indicated the mitochondrial protein interactions of TFAM and TFB2M as a strong and independent prognostic factor for 30-day survival (**Table 3**).

**Table 3:** Cox regression analysis in patients with critical COVID-19 regarding 30-day mortality.

	Univariable		Multivariable	
	*Hazard ratio (HR)*		*Adjusted Hazard Ratio (aHR)*	
Covariable	(95 % CI)	p-value	(95 % CI)	p-value
TFAM-TFB2M* [interactions per cell]	3.7 (1.9 to 7.2)	< 0.001	3.3 (1.6 to 6.5)	<0.001
Age			1.06 (0.99 to 1.14)	0.12
SOFA-Score			1.01 (0.99 to 1.04)	0.24

Hazard Ratio point estimates, 95 % CI, and p-values (two-sided) are reported; *Mitochondrial protein activity described by the proximity ligation assay of TFAM and TFB2M expressed as interactions per cell. The adjusted Hazard Ratio (aHR) is corrected for the covariates age and SOFA score.

Additionally, we observed that the group with reduced TFAM-TFB2M interactions (≤10.7 PLA signals per cell) had a median of 0 ICU-free days [IQR: 0 to 3] over a 60-day period, in contrast to 28 ICU-free days [IQR: 4 to 25] for the group with increased TFAM-TFB2M interactions (> 10.7 PLA signals per cell; p < 0.001; **Figure 3B**).

## 4 Discussion

Our study unveils mitochondrial protein interactions as a novel biomarker in critical COVID-19. Specifically, we shed light on the promising role of mitochondrial biogenesis assessed via the interaction between TFAM and TFB2M in peripheral blood of critical COVID-19 patients. Remarkably, compared to mRNA transcripts of MQC-related genes and the mtDNA copy number, the TFAM-TFB2M mitochondrial protein interaction exhibited superior capability in reflecting disease severity and was correlated with clinical outcomes. Specifically, enhanced mitochondrial biogenesis, indicated by elevated TFAM and TFB2M interactions on day 1, was associated with a 74% survival rate at 30 days and a greater number of ICU-free days. In contrast, patients with decreased interactions of TFAM and TFB2M had more than 3-fold higher risk to die within the first 30 days. These results highlight the promising potential of TFAM-TFB2M protein interactions as a new and clinically useful biomarker for mitochondrial dysfunction in severe COVID-19.

One important question is why the TFAM-TFB2M mitochondrial protein interaction stands out as a novel biomarker superiorly reflecting clinical outcome of critical COVID-19, compared to other mRNA-based biomarkers, which also target processes of MQC. MQC involves balancing mitochondrial synthesis, remodeling, and degradation to repair damage effectively. Failure in these MQC processes correlates with worse outcomes (21). Studies highlight the role of mitochondrial biogenesis in recovery from COVID-19, positioning MQC as a critical tool for evaluating and predicting recovery from organ failure (14, 15). In this context, several recent studies detected a higher amount of cell-free mtDNA (cf-mtDNA) in patients with severe COVID-19 compared to asymptomatic COVID-19 patients, indicating that elevated mtDNA levels may be correlated with COVID-19 poor outcomes (21, 22). These results are contrary to our findings, as in our study, we did not find a difference in mtDNA copy number between survivors and non-survivors of COVID-19. However, this is mainly explained by the fact, that both Edinger et al. and Hepokowski et al. quantified mtDNA in plasma, i.e. cf-mtDNA, whereas our measurements of mtDNA copy numbers were performed on whole blood, mainly encompassing the mtDNA content of cells (21, 22). In this context, assessing cf-mtDNA is a surrogate for the associated tissue damage rather than a marker of MQC (23, 24). Of note Valdes and colleagues indeed demonstrated a higher amount of mtDNA copy number in whole blood, so focusing MQC, of COVID-19 patients with mild to moderate symptoms compared to critically ill COVID-19 patients and non-survivors (12). Although we cannot confirm this finding, the cellular mtDNA content in blood cells may also represent a biomarker for mitochondrial repair in critical COVID-19.

Regarding MQC-related genes, we observed that mRNA transcripts of these genes are not associated with outcome of our critically ill COVID-19 patients. Here, numerous studies have demonstrated upregulation of gene transcripts involved in MQC in COVID-19 (25, 26). Shokora et al. examined the mRNA expression of TFAM in COVID-19 and demonstrated elevated levels of TFAM gene expression in COVID-19 patients compared to controls (27), which is at least counterintuitive that patients with an enhanced mitochondrial biogenesis suffer from severe infection. In our previous study, we observed a protein maldistribution of TFAM in septic patients as potential explanation (16). Here, an increased amount of TFAM was detected in the cytosol whereas a decreased concentration of TFAM was found in the mitochondria (16). In this context, it is important to note that TFAM is expressed in the nucleus, translated in the cytosol, and subsequently actively transported to the mitochondria (28, 29). Thus, an elevated cytosolic mRNA and protein concentration of TFAM, not necessarily transposes in effective mitochondrial biogenesis during severe bacterial and viral infections, especially in sepsis. This protein maldistribution of TFAM could explain the observed diverge between TFAM mRNA expression and TFAM protein activity in sepsis (19) and now in this study in patients with COVID-19 and also likely include other nuclear-encoded mitochondrial proteins. Consequently, assessing TFAM’s functional activity provides more advanced “down-stream” approach for predicting clinical recovery. Here, our study introduces a new method to evaluate the interaction between the mitochondrial proteins TFAM and TFB2M, overcoming the limitations of current mRNA and protein biomarkers in COVID-19. Interestingly, our previous multicentric study could already confirm the TFAM-TFB2M interaction as a new biomarker, indicating mitochondrial recovery, in bacterial sepsis (19). Here, it was determined that this interaction serves as a precise indicator of disease severity and predicts clinical outcomes of septic patients as well. Thus, the mitochondrial biogenesis seems to be pivotal for recovery from bacterial sepsis as well as from critical COVID-19.

Since mitochondrial biogenesis seems to indicate disease severity and reflect organ dysfunction, an additional assessment of the affected organs would have been desirable. Thus, skeletal muscle biopsy studies have significantly contributed to our understanding of mitochondrial structure and function changes during sepsis and COVID-19 (28–31). However, these examinations are challenging due to the invasiveness of muscle biopsies and the time and effort required. Consequently, there is a growing interest in noninvasive tests using peripheral blood to provide information about a patient’s overall mitochondrial health. Assessing highly aerobic human PBMCs is particularly suitable for reflecting the mitochondrial function of other aerobic, adenosine triphosphate (ATP)-consuming organs such as the brain, heart, kidneys, liver, and lungs, which are susceptible to mitochondrial damage (15, 32). Therefore, the mitochondria of PBMCs likely serve as a useful proxy for the overall mitochondrial state in COVID-19 (9, 33, 34). Of note, the protein interaction between TFAM and TFB2M is just one promising example among many significant protein interactions involved in severe infections like critical COVID-19. Strikingly, our study demonstrates that assessing protein interactions can address major limitations of mRNA or protein biomarkers. Consequently, our work represents a crucial milestone that paves the way for innovative biomarkers in critical COVID-19. By using a proximity ligation assay to examine the interaction between TFAM and TFB2M, we were able to assess the mitochondrial activity of these proteins *in situ* with remarkable reliability (35).

Furthermore, despite the notable differences between bacterial sepsis and COVID-19, such as the immune response, we have been able to illuminate key shared pathophysiological mechanisms, for instance mitochondrial dysfunction and potential mislocalization of TFAM, underlying both conditions. Thus, organ dysfunction, characterized by mitochondrial dysfunction, and mitochondrial biogenesis, as hallmark of mitochondrial recovery, might represent a common pathomechanistical final stage of both, bacterial sepsis and COVID-19.

The limitations of this study are as follows: First, our study may introduce a selection bias, as we solely recruited on university ICUs and there potentially selected particular vulnerable patients. Second, we just examine the novel biomarker at one timepoint. Further studies are needed to investigate the TFAM-TFB2M-interatction during the course of COVID-19. Third, we did not differentiate among PBMC subpopulations. Thus, we cannot dismiss the possibility of varying contributions from different cell populations, potentially due to differences in mitochondrial content. Fourth, various factors impact on TFAM-TFB2M protein interactions, such as changes in mtDNA copy number, mitochondrial membrane potential, or import disorders of the transcription factors, which could impede accurate biological interpretations (36). Further studies will be needed to validate our findings and elaborate on the pathomechanistical role of mitochondrial biogenesis in COVID-19.

In conclusion, our findings highlight the importance of the TFAM-TFB2M protein interaction as a strong and independent predictor of 30-day survival in critical COVID-19. Strikingly, this potential novel biomarker targeting mitochondrial biogenesis is measurable in peripheral blood and does not require any invasive diagnostic procedure. In addition, with these findings we could elucidate a common pathomechanistical final stage of both, bacterial sepsis and COVID-19.

## Data Availability

The original contributions presented in the study are included in the article/supplementary material. Further inquiries can be directed to the corresponding author/s.
